# Long-term platinum retention after treatment with cisplatin and oxaliplatin

**DOI:** 10.1186/1472-6904-8-7

**Published:** 2008-09-17

**Authors:** Elke EM Brouwers, Alwin DR Huitema, Jos H Beijnen, Jan HM Schellens

**Affiliations:** 1 Department of Pharmacy & Pharmacology, Slotervaart Hospital/The Netherlands Cancer Institute, Louwesweg 6, 1066 EC Amsterdam, The Netherlands; 2 Beta faculty, Department of Pharmaceutical Sciences, Division of Biomedical Analysis, Section of Drug Toxicology, Utrecht University, P.O. Box 80082, 3508 TB Utrecht, The Netherlands; 3 Department of Medical Oncology, Antoni van Leeuwenhoek Hospital/The Netherlands Cancer Institute, Plesmanlaan 121, 1066 CX Amsterdam, The Netherlands

## Abstract

**Background:**

The aim of this study was to evaluate long-term platinum retention in patients treated with cisplatin and oxaliplatin.

**Methods:**

45 patients, treated 8–75 months before participating in this study, were included. Platinum levels in plasma and plasma ultrafiltrate (pUF) were determined. In addition, the reactivity of platinum species in pUF was evaluated. Relationships between platinum retention and possible determinants were evaluated.

**Results:**

Platinum plasma concentrations ranged between 142–2.99 × 10^3 ^ng/L. Up to 24% of plasma platinum was recovered in pUF. No platinum-DNA adducts in peripheral blood mononuclear cells (PBMCs) could be detected. *Ex vivo *incubation of DNA with pUF of patients revealed that up to 10% of the reactivity of platinum species was retained. Protein binding proceeded during sample storage. Sodium thiosulfate (STS) appeared to release platinum from the plasma proteins. Platinum levels were related to time, dose, STS co-administration, and glomerular filtration rates (GFR).

**Conclusion:**

Our data suggest that plasma platinum levels are related to time, age, dose, GFR, and STS use. Platinum in plasma, probably, represent platinum eliminated from regenerating tissue. Platinum species in pUF were partly present in a reactive form. The effects of the reactivity on long-term consequences of Pt-containing chemotherapy, however, remains to be established.

## Background

Since its discovery as an effective anticancer agent in the 1960s [1], cisplatin is used extensively in oncology. The use of platinum (Pt) agents has had an enormous impact on the prognosis of several cancer types. After the introduction of cisplatin, mortality of e.g. testicular cancer reduced significantly. Also oxaliplatin has found a widespread use in the treatment of cisplatin resistant colorectal cancer [2].

The improved life expectancy of cancer patients treated with Pt-based compounds, has led to an increased interest in the long-term side effects of these drugs, such as peripheral neuropathy, nephrotoxicity, and ototoxicity [3-6]. The presence of long-term side effects has led to the investigation of long-term pharmacokinetics, distribution, and elimination of Pt-based drugs. Studies have shown that with a standard cisplatin-containing chemotherapy, plasma and tissue Pt levels are still remarkably elevated years after chemotherapy [7-12]. For oxaliplatin, no data on long-term pharmacokinetics are available yet. In addition, no studies have been performed to investigate the potential reactivity of retained Pt species years after treatment.

In the current study the long-term Pt retention in plasma and plasma ultrafiltrate (pUF) of patients treated with cisplatin or oxaliplatin up to 6 years before participating in this study was investigated. The *in vivo *reactivity of circulating Pt was studied by testing the DNA- and protein binding activity of ultrafilterable Pt and the ability of sodium thiosulfate (STS) to release Pt from the plasma proteins. For quantification of Pt levels in plasma, pUF, and for quantification of the level of Pt-DNA adducts, we used inductively coupled plasma mass spectrometry (ICP-MS). Finally, potential relationships between Pt exposure and follow-up time, age, cumulative dose, route of administration, renal function, glutathione S-transferase (GST) genotypes, and co-administration of STS with intra-arterial cisplatin were investigated.

## Methods

### Participants

For cisplatin, patients were selected at random from all patients who started treatment between 2000 and 2004, received cumulative cisplatin doses of ≥ 300 mg/m^2^, and were available for follow-up. This was done to obtain a heterogeneous sample from the population of patients treated with cisplatin. For this pilot study, 20 patients of the 400 eligible patients were included. To select the patients, random selections were performed on the 400 eligible patients until 20 patients agreed to participate in the study. SPSS (SPSSinc, version 11.0, Chicago, IL, USA) was used for random sample selection. Unfortunately, for oxaliplatin, no random selection could be performed because, the number of available patients was too low. This was due to a high mortality rate of the patients treated with oxaliplatin. Therefore, for oxaliplatin, all available patients who started treatment between 2000 and 2005 and received cumulative oxaliplatin doses of ≥ 600 mg/m^2 ^were approached for participation in the current study. This led to an inclusion of 25 patients. The Medical Ethics Committee of the hospital approved the study protocol and all patients gave their written informed consent.

Additionally, 20 cancer patients who were not treated with cisplatin and 20 healthy volunteers, were included as a control for Pt background levels in plasma.

### Blood sampling

Whole blood samples for Pt analysis were collected in 10 mL EDTA containing tubes (Becton Dickinson Vacutainer Systems, Plymouth, UK). EDTA was preferred above heparin because the fraction peripheral blood mononuclear cells (PBMCs) could be isolated easier from EDTA plasma. No difference in the ultrafiltrable fraction was observed between EDTA or heparin containing tubes. Plasma was obtained by centrifuging the whole blood samples for 15 min (1,000 g, 4°C). The fraction containing PBMCs was isolated from the whole blood sample using the method described by Pluim et al [13]. PUF was obtained by centrifuging the plasma fraction through 3 and 30 kDa cut-off ultrafiltrate filter (Centriplus Millipore Corporation, Bedford, MA, USA) for 30 min (1,000 g, 20°C). Additionally, from each patient, 5 ml blood samples were obtained for genetic analysis. Lymphocyte DNA was isolated according to the method of Boom [14]. All samples were stored at -20°C until analysis.

### Determination of Pt levels

Pt analyses were performed using an ICP-MS and a validated method described previously [15]. Pt levels were assessed in plasma, pUF, and bound to DNA in PBMCs. DNA was isolated from PBMCs using a method described by Pluim et al [13]. Before ICP-MS analysis, the DNA was hydrolysed in 1% *(v/v) *HNO_3 _solution at 70°C. The limit of quantification (LLOQ) of the method was 20 ng/L for plasma, 7.5 ng/L for pUF, and the absolute sensitivity for Pt bound to DNA was 0.75 pg Pt (7.5 fg Pt per μg DNA when using 100 μg DNA). The LLOQs were determined on the basis of five times the noise in blank matrix solutions.

### Assessment of DNA binding activity of Pt in pUF

PUF samples (500 μL) which contained high Pt concentrations of two cisplatin and two oxaliplatin treated patients, were incubated with an excess of calf thymus DNA (500 μg) (Sigma-Aldrich) to allow maximal binding. Additionally, pUF from healthy volunteers was incubated with 500 μg of calf thymus DNA and cisplatin or oxaliplatin with Pt concentrations equivalent to the investigated patients samples. The latter was done to assess the binding capacity of the parent compounds at concentrations in the same range as the patients samples. After a five-day incubation to achieve a maximal Pt-DNA binding, the DNA was precipitated, washed and dissolved in water as described by Brouwers et al [16]. After hydrolysis, the Pt-DNA binding was assessed.

### Ex vivo assessment of protein binding capacity of Pt in pUF

To assess the protein binding capacity of Pt, pUF was prepared for four cisplatin and oxaliplatin plasma samples after storage at -30°C for 144–278 days. Pt concentrations analysed in pUF prepared after storage were compared to Pt concentrations analysed in pUF which was prepared immediately after blood sampling. Additionally, plasma samples were reanalyzed to assess whether or not Pt concentrations in plasma were reduced during storage due to adsorption to the tubes.

### Ex vivo activity of STS

Four plasma samples for both cisplatin and oxaliplatin were used to investigate the ability of STS to remove Pt from proteins. Therefore, 750 μL of plasma were incubated with 25 μL of a 250 g/L STS solution. As control, 750 μL of plasma were incubated with 25 μL of water. After incubation, pUF was prepared and Pt concentrations of the STS incubated samples and control samples were compared. Protein concentrations in the ultrafiltrates were analysed using a 2-D Quant Kit (GE healthcare Bio-Sciences AB, Uppsala, Sweden).

Two pUF samples (500 μL) which contained high Pt concentrations of one cisplatin and one oxaliplatin treated patient, were incubated with an excess of calf thymus DNA (500 μg) (Sigma-Aldrich) in duplicate. Pt-DNA binding was analysed with and without incubation of STS.

### Genotyping

Polymorphisms in the genes encoding the enzymes *GSTM1*, *GSTT1*, and *GSTP1 *were determined. In *GSTT1 *and *GSTM1*, known inherited homozygous deletions are equivalent to nonfunctional enzymes [17]. In the *GSTP1 *gene, a functional SNP between adenosine (A) and guanosine (G) at base pair 313 leads to the expression of either Ile or Val at codon 105. This polymorphism significantly affects enzyme activity [18].

*GSTM1 *and *GSTT1 *deletions were analysed using a gel electrophoresis method as described by Sreelekha et al [19]. *GSTP1 *(exon 5) was genotyped according to Jerónimo et al [20].

### Clinical parameters

Information regarding cumulative cisplatin and oxaliplatin dose, follow-up time (time since end of treatment), route of Pt administration, co-administration of STS, and serum creatinine before start of chemotherapy were collected from patient files. Additionally, serum creatinine was assessed at the time of study.

### Statistical analyses

Differences between Pt levels of cisplatin and oxaliplatin treated patients, control cancer patients, and healthy controls were evaluated using the Mann-Whitney *U *test. The Wilcoxon signed rank test was used to test the difference between the renal function at the time of chemotherapy and at follow-up. Correlations between plasma and pUF level of Pt treated patients were evaluated by the Pearson correlation coefficient. SPSS was used to perform these tests.

Possible relationships between determinants and Pt levels were evaluated using non-linear mixed effects modeling using NONMEM software (Version V1) (GloboMax LLC, Ellicott city, MD, USA). The first order conditional estimation method was used throughout. It was assumed that, due to the long follow-up, the treatment period was negligible compared to the follow-up time. The significance of established relationships was assessed using the likelihood ratio test.

## Results

### Participants

Table 1 summarizes the characteristics of the participants. Participants were treated with cisplatin for diverse tumour types, whereas all patients treated with oxaliplatin were diagnosed with colorectal cancer. The range in the follow-up time of patients was between 8 and 75 months.

**Table 1:** Characteristics of participants

	**Cisplatin**	**Oxaliplatin**
Gender (m/f)	13 m/7 f	20 m/5 f
Age at time of follow-up (median)	49 years	64 years
Duration of follow-up	18–75 months (median 41)	8–33 months (median 18)
Tumour type	Testicular carcinoma (9)	Colorectal carcinoma (25)
	Yolk sac carcinoma (1)	
	Non small cell lung cancer (1)	
	Small cell lung cancer (1)	
	Head and neck carcinoma (8)	
Cumulative dose	300–600 mg/m^2 ^cisplatin (median 350)	585–1170 mg/m^2 ^oxaliplatin (median 878)
	195–390 mg/m^2 ^Pt	287–575 mg/m^2 ^Pt
	(median 227)	(median 431)
Sodium thiosulfate	5 head and neck carcinoma patients treated intra-arterially with 600 mg/m^2 ^cisplatin	NA
Ca/Mg infusion	NA	24/25
GSTM1	8/20 positive, 12/20 negative	10/25 positive, 15/25 negative
GSTT1	17/20 wildtype, 3/20 negative	21/25 positive, 4/25 negative
GSTP1	12/20^105^Ile/^105^Ile-*GSTP1*	9/25^105^Ile/^105^Ile-*GSTP1*
	7/20^105^Val/^105^Ile-*GSTP1*	10/25^105^Val/^105^Ile-*GSTP1*
	1/20^105^Val/^105^Val-*GSTP1*	6/25^105^Val/^105^Val-*GSTP1*

NA = not applicable

### Pt levels

**Figure 1:**
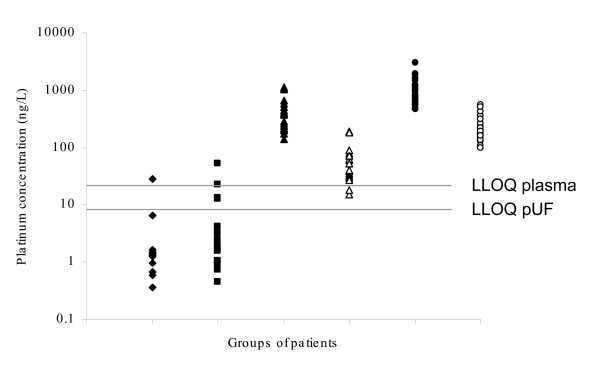
Pt concentrations of 20 healthy controls (plasma ◆), 20 cancer control patients (plasma ■), 20 cancer patients who were treated with cisplatin 18–73 months before entering this study (plasma ▲ and pUF △), 25 cancer patients who were treated with oxaliplatin 8–23 months before entering this study (plasma ● and pUF ○).

Figure 1 shows Pt levels in plasma of 20 healthy controls, 20 cancer control patients, 20 cisplatin treated cancer patients treated 18–75 months before entering this study, and 25 oxaliplatin treated cancer patients treated 8–33 months before entering this study. Moreover, pUF levels from Pt treated patients are shown. All plasma and pUF samples of the Pt treated patients were above the LLOQ of the method (plasma: 142–2.99 × 10^3 ^ng/L median 647 ng/L, pUF: 15.3–565 ng/L, median 157 ng/L), whereas only three plasma samples of control patients exceeded the LLOQ (plasma: < LLOQ-55.6 ng/L). Pt concentrations in plasma of Pt treated patients were significantly higher than those in control patients (Mann-Whitney *U *test, p < 0.0001). Pt levels in pUF were highly correlated to levels in plasma. The Pearson correlation coefficients were 0.97 and 0.95 for cisplatin and oxaliplatin respectively. For cisplatin, on average 14.8% of plasma Pt was recovered in pUF. This percentage was 24.2% for oxaliplatin. The percentage of Pt in pUF was not dependent on the amount of Pt in plasma. No difference was observed between 3 and 30 kDa filters. Levels of Pt-DNA adducts in PBMCs were below LLOQ of 0.75 pg Pt in all samples.

### Assessment of DNA binding activity of Pt in pUF

Cisplatin and oxaliplatin added to pUF and incubated *ex vivo *with DNA revealed that after five days, for both compounds, 21% of the added Pt was bound to DNA. The pUF samples of the cisplatin patients demonstrated a DNA binding of 1.8 and 2.4%. For oxaliplatin, these percentages were 0.78 and 1.1%. Hence, the experiment showed that for cisplatin and oxaliplatin, on average, respectively 10 and 4.3% of the total binding capacity of equivalent concentrations of parent compound was recovered more than 8 months after the end of treatment. Pt contents of the DNA samples incubated with the patient samples were just above the LLOQ of the method (2.1–6.0 pg).

### Ex vivo assessment of protein binding capacity of Pt in pUF

Table 2 shows the results of the experiments in which the plasma protein binding capacity of Pt in plasma samples of patients was investigated. On average, the decrease in Pt concentrations in pUF was 45% and 26% for cisplatin and oxaliplatin, respectively after storage for 144–278 days. For both compounds, plasma Pt concentrations remained constant over time.

**Table 2:** Pt concentrations in eight pUF samples before and after storage

**Compound**	**Percentage of plasma Pt recovered in pUF before storage**	**Percentage of plasma Pt recovered in pUF after storage**	**Days of storage**
Cisplatin	19.4	8.1	278
	19.7	8.4	278
	15.1	5.5	276
	12.3	7.4	276
Oxaliplatin	27.8	21.2	180
	25.9	16	161
	22.9	15.8	157
	28.2	25.2	144

### Ex vivo activity of STS

The results for the Pt concentrations in pUF samples with and without prior addition of STS to plasma are depicted in Table 3. For cisplatin and oxaliplatin, respectively, a 2.3- and 1.6-fold increase in Pt pUF concentrations was observed in samples prepared from STS treated plasma. No proteins could be detected in any of the pUF samples.

**Table 3:** Pt concentrations in eight pUF samples with and without incubation with STS

**Compound**	**Percentage of plasma Pt recovered in pUF**	**Percentage of plasma Pt recovered in pUF after STS incubation**	**Factor increase of Pt in pUF after STS incubation**
Cisplatin	12.4	17.5	1.4-fold
	12.7	34.9	2.7-fold
	13.2	30.7	2.3-fold
	12.5	36.6	2.9-fold
Oxaliplatin	32.2	46.2	1.4-fold
	20	39.5	2.0-fold
	26.5	38.7	1.5-fold
	27.2	40.4	1.5-fold

The effect of STS on the Pt-DNA binding in the pUF samples could not be established.

### Effects of determinants on in vivo plasma Pt levels

**Figure 2:**
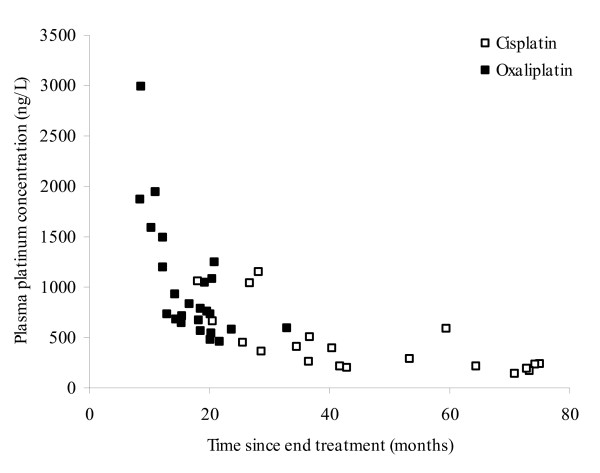
Plasma Pt concentrations versus time since end of treatment.

Plasma Pt levels showed a gradual decline over follow-up time (Figure 2). The decrease of the Pt levels followed a first order elimination profile. The elimination profiles could be desribed by two-compartment models for both compounds. Two-compartment models were superior to one compartment models. The first elimination half-life (t_1/2_) for cisplatin was 5.02 months and the second 37.0 months. For oxaliplatin, these half-lifes were 1.37 and 535 months. The values of the first phase of cisplatin and the second phase of oxaliplatin, however, had relatively high standard errors. Because of the observed overlap of follow-up time for cisplatin and oxaliplatin (18–75 months vs 8–23 months) the data were also combined. The combined elimination profile could be described by a two-compartment model of which the first elimination half-life (t_1/2_) was 1.2 months and the second t_1/2 _28.5 months. The second t_1/2 _was related to age, in which a twofold increase in age resulted in a 1.4-fold longer t_1/2 _(p = 0.002). Combination of the data led to more reliable parameter estimates of the two compartment model because the time window was larger compared to the analyses of the compounds separately. Evidently, we should take into account that the first half life was mostly determined by oxaliplatin data and the second half life was mostly determined by cisplatin data.

**Figure 3:**
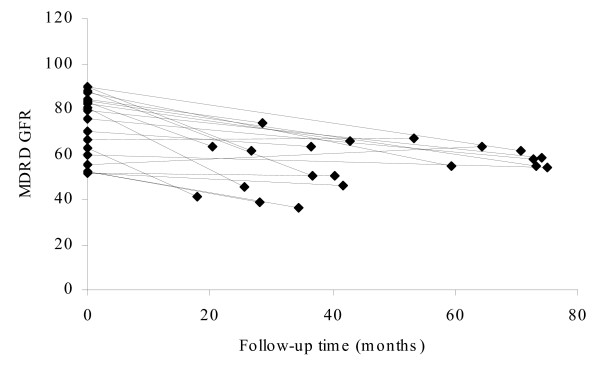
GFR values for cisplatin treated patients at time of treatment (t = 0) and at follow-up.

**Figure 4:**
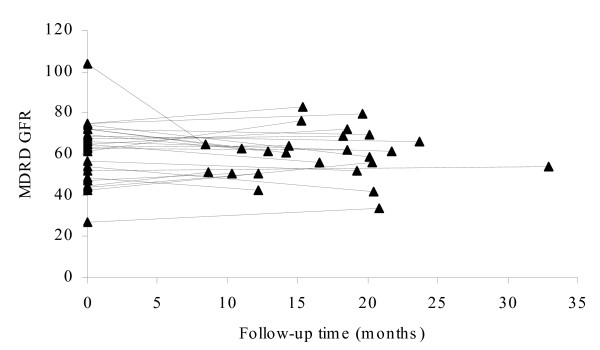
GFR values for oxaliplatin treated patients at time of treatment (t = 0) and at follow-up.

Pt levels were proportional to the cumulative dose. STS co-adminstration in combination with intra-arterial cisplatin administration led to a 71% reduction in Pt levels (p < 0.001). An association between *GSTT1*,*GSTM1*, and *GSTP1 *genotypes and Pt levels could not be established. The effect of the renal function on Pt levels was evaluated using the glomerular filtration rate (GFR) which was calculated with the 'Modification of Diet in Renal Disease (MDRD)'-formula [21]. Median GFR values before start of the Pt chemotherapy were 78 and 65 mL/min/1.73 m^2 ^for cisplatin and oxaliplatin, respectively. At the time of the current study, GFR values of cisplatin patients were significantly decreased to 55 mL/min/1.73 m^2 ^(p < 0.001), whereas GFR values for oxaliplatin patients remained constant (61 mL/min/1.73 m^2^). Figure 3 and 4 show the MDRD GFR for patients at the start of their chemotherapy treatment and at follow-up. The GFR at the time of chemotherapy was significantly and conversely related to plasma Pt levels (p < 0.01). Long-term plasma Pt concentrations were lower when the GFR at the time of chemotherapy was higher.

## Discussion

Since the discovery of the antineoplastic effects of Pt-based compounds, cisplatin and later oxaliplatin have developed into commonly used anticancer agents. The increased survival of patients treated with these agents and the associated long-term side effects, have initiated the investigation of the long-term pharmacokinetics, distribution, and elimination of Pt-based compounds.

The current study focussed on the long-term pharmacokinetics of cisplatin and oxaliplatin in plasma and pUF. We showed that plasma Pt levels of patients, treated with cisplatin or oxaliplatin 8 to 75 months before participating in the current study, were > 30-fold higher than the mean level of unexposed controls. In earlier studies raised plasma or serum Pt levels until 240 months after treatment with cisplatin were also reported [7,8,10,11].

In addition to elevated plasma Pt levels, the ultrafiltrable fraction of the plasma also contained Pt species. This has not been reported before. The fraction of plasma Pt recovered in the pUF was higher for oxaliplatin than for cisplatin, which could be a result of a higher reactivity of cisplatin and hence, a more extensive protein binding of cisplatin. Because pUF is generally considered to contain the pharmacologically active Pt fraction [22] and inactive species [23], the question arises as to whether ultrafiltrable Pt measured up to 75 months after chemotherapy is composed exclusively of inactive Pt bound to low-molecular-weight molecules smaller than 3 kDa, or whether it might also contain bound or unbound Pt with retained reactivity. Because ICP-MS can not distinguish between unchanged cisplatin/oxaliplatin and its metabolites or adducts, no information on the composition of the Pt species in the pUF samples could be obtained. Unfortunately, up to now, no other technique is sensitive enough to elucidate the chemical composition of the pool of Pt metabolites and adducts that are probably present in the pUF samples.

Therefore, to address the question whether the Pt present in the pUF samples was still reactive, we attempted to assess the Pt-DNA binding activity *in vivo *and *ex vivo*. We were not able to quantify Pt-DNA adduct levels in PBMCs of the patients. This observation, however, does not mean that there are no Pt-DNA adducts present. The Pt concentrations in pUF were only 2 to 75 fold higher than the LLOQ in this matrix. Therefore, Pt-DNA adduct levels in PBMCs might well be under the LLOQ of the method of 7.5 fg Pt/μg DNA [16]. Although we were not able to quantify Pt-DNA adduct levels *in vivo*, we did show that for the highest concentrated cisplatin and oxaliplatin patient pUF samples, respectively 10% and 4.3% of the DNA binding activity of the parent compound was retained. The difference between the reactivity of cisplatin and oxaliplatin samples is in agreement with the difference in DNA binding activity of the parent compounds. [24,25]. Whether the Pt-DNA adducts are similar to the adducts formed by the parent compounds remains to be established. Unfortunately, the DNA binding activity could only be evaluated in the highest concentrated pUF samples. It is relevant to consider that these samples might not be representative for all pUF samples.

In addition to the remaining DNA binding properties, Pt in plasma also appeared to have remaining protein binding capacity, which substantiates the observations that Pt species recovered years after treatment may still show reactivity. The reduction in ultrafilterable Pt of 45% and 26% for cisplatin and oxaliplatin, respectively after storage for 144–278 days was less than the reduction observed by Erkmen et al. [26] for carboplatin after storage for 100 days. This difference might be caused by the difference in Pt concentration of the samples (ng/L in the current study, versus mg/L in the study described by Erkmen et al.) or by the difference in storage temperature (-30°C for the current study, versus -20°C in the study described by Erkmen et al.). Furthermore, the samples of Erkmen were incubated in vitro with carboplatin, whereas the samples of the current study were obtained from patients up to years after treatment. The activity of the circulating Pt, therefore, might be lower in the current sample set, leading to less reduction of Pt in pUF after storage.

The investigation of the Pt levels in pUF after the *ex vivo *incubation of plasma with STS revealed that STS incubation resulted in higher Pt levels in pUF. This suggests that at least part of the Pt protein binding is not irreversible and that a nucleophilic compound such as STS is capable of releasing Pt from proteins because the nucleophilic sulfide group, possibly, has a higher binding affinity for Pt than proteins do. This observation, however, should be interpreted with caution because the effect of STS on protein structure and thus of the ability of the proteins to pass the ultrafiltration membrane could not be evaluated because protein concentrations in pUF were too low to be detected. The effect of STS on the Pt-DNA adducts, could not be established.

Investigation of the effects of determinants on plasma Pt levels revealed a strong relationship between Pt levels and the follow-up time. The relationship suggested that plasma Pt was eliminated according to a first order elimination profile, of which the first t_1/2 _(1.2 months) could be estimated accurately for oxaliplatin and the second t_1/2 _(28.5 months) could be estimated for cisplatin. For cisplatin, associations between plasma Pt levels and follow-up time were published before [7-11]. Hohnloser et al. reported an elimination half-life of 6.6 months for the time segment of 5.4 to 32 months and 26 months for the time segment of 21 to 107 months [11]. The last t_1/2 _is in agreement with our findings. Although the two elimination half-lives found in this study characterize the data between 8 and 75 months after the end of treatment, the complete elimination of plasma Pt can presumably be described by numerous half-lives which increase with a longer follow-up period. This was confirmed by investigations of Gelevert et al., who calculated a t_1/2 _of 54 months for patients who were treated with cisplatin 120 to 240 months before follow-up.

The long-term Pt plasma levels are, most probably, a result of Pt accumulation which is released into the bloodstream due to regeneration of tissue. Several investigations have shown raised Pt levels in tissue samples up to years after chemotherapy [27,28]. The two compartments in the Pt elimination observed in this study, could be explained by the Pt release from fast regenerating tissue, followed by a release from slower regenerating tissue. This hypothesis is supported by observations of Heydorn et al. and Gregg et al. Heydorn et al. who reported that different tissues showed variable elimination half-lives. Gregg et al. even showed that Pt levels in peripheral nerve tissue such as the dorsal root ganglia, did not decay with time [28], which was expected considering the slow regeneration of peripheral nerve tissue [29]. Because the rate of regeneration of tissue decreases with age, the observation that a higher age was associated with a longer t_1/2 _was in correspondence with the hypothesis that Pt levels recovered in the plasma represent the regeneration of tissue.

In addition to follow-up time, the cumulative dose also appeared to be associated with plasma Pt levels, which was expected as a higher initial Pt load will result in higher tissue concentrations [28] and thus higher long-term plasma Pt levels. Few other studies also suggested a correlation between cumulative Pt dose and long-term plasma or serum Pt levels [8,11].

The association between renal function and plasma Pt levels was also evaluated. In a previous study it was shown that urinary Pt concentrations of patients studied 5.3 to 16.8 years after completion of cisplatin chemotherapy, were strongly correlated to serum concentrations suggesting rate limiting release of Pt from the tissues followed by fast renal excretion [10]. This observation implies that no effect of renal function at follow-up on plasma Pt elimination would be expected. The renal function before start of the treatment, however, could affect the initial elimination of Pt from pUF [30] and thus the total level of Pt accumulation in the tissues. Therefore, renal function might affect long-term plasma Pt levels. This hypothesis was in accordance with our observation that a higher GFR at the time of treatment was associated with lower plasma Pt levels.

A similar approach counts for the administration of STS at the time of chemotherapy. The binding of STS to cisplatin could inactivate cisplatin resulting in a reduction of initial Pt accumulation in tissue. Our results demonstrated that long-term plasma Pt levels were reduced by 71% with co-administration of STS, which is in agreement with a reduced tissue accumulation. Although, the intra-arterial administration of cisplatin in patients who received STS could affect the venous plasma pharmacokinetics after administration due to a first pass effect in tissue [31], we do not expect that the administration route affects the total amount of Pt bound to tissue and thus the long-term plasma Pt levels. The first-pass extraction by the tissue after i.a. administration might lead to an increased instantaneous accumulation of Pt in the tissue. This does not increase the elimination of Pt from the body, as STS does. In contrast, i.a. administration might increase the long-term Pt levels because of the initial increase of Pt retention in the tissues. A definite conclusion concerning this matter, however, cannot be drawn as there are no studies available describing the difference between i.a. administered equal doses of cisplatin with or without co-administration of STS.

Although no reports have been published on the effects of GST genotypes on Pt pharmacokinetics, GST genotypes could, considering the detoxification mechanism for Pt from cells [32], affect the initial Pt elimination from the tissues and thus the long-term tissue and plasma Pt levels. For the current study, however, no such relationship could be established, possibly due to a low number of patients.

## Conclusion

Our data suggest that plasma Pt levels are related to follow-up time, age, cumulative dose, GFR at time of treatment, and STS use. Although no Pt-DNA adducts could be detected in PBMCs, it was shown that Pt species in pUF were still present in a reactive form. The effects of the observed reactive species on long-term consequences of Pt-containing chemotherapy, however, remains to be established.

## Abbreviations

pUF: plasma ultrafiltrate; PBMCs: peripheral blood mononuclear cells; STS: sodium thiosulfate; GFR: glomerular filtration rate; Pt: platinum; ICP-MS: inductively coupled plasma mass spectrometry; GST: glutathione S-transferase; LLOQ: limit of quantification; MDRD: modification of diet in renal disease

## Competing interests

The authors declare that they have no competing interests.

## Authors' contributions

EB: participated in the design of the study, performed the study, performed the data analysis, and drafted the manuscript. AH: participated in the design of the study, data analysis and performed the statistical analysis. JB: participated in the design of the study and data analysis. JS: participated in the design of the study and data analysis.

## Pre-publication history

The pre-publication history for this paper can be accessed here:


